# P-2337. Contemporary Epidemiology of Infant Mortality Attributable to Cytomegalovirus Infection in the United States, 2007-2021

**DOI:** 10.1093/ofid/ofae631.2489

**Published:** 2025-01-29

**Authors:** Ladonna Boasiako, Ngozika G Obiefuna, Abigail Arthur, Chidinma S Ononogbu, Fredrick Dapaah-Siakwan

**Affiliations:** No Affiliation, Brandywine, Maryland; University of Abuja Teaching Hospital, Nigeria., katy, Texas; Johns Hopkins School of Medicine , Department of Pediatric Infectious Diseases, Baltimore, Maryland; Korle Bu Teaching Hospital, Accra, Accra, Greater Accra, Ghana; Valley Children's Pediatric Residency Program, Madera, California

## Abstract

**Background:**

Cytomegalovirus (CMV) infections are common in infants in the United States (US) but infant mortality due to CMV has been poorly studied and it is unknown if there have been changes in CMV infant mortality rate (CMV-IMR) over time. We described the characteristics of a contemporary national cohort of infants who died from CMV and whether the CMV-IMR changed from 2007 to 2021.

**Table 1.**
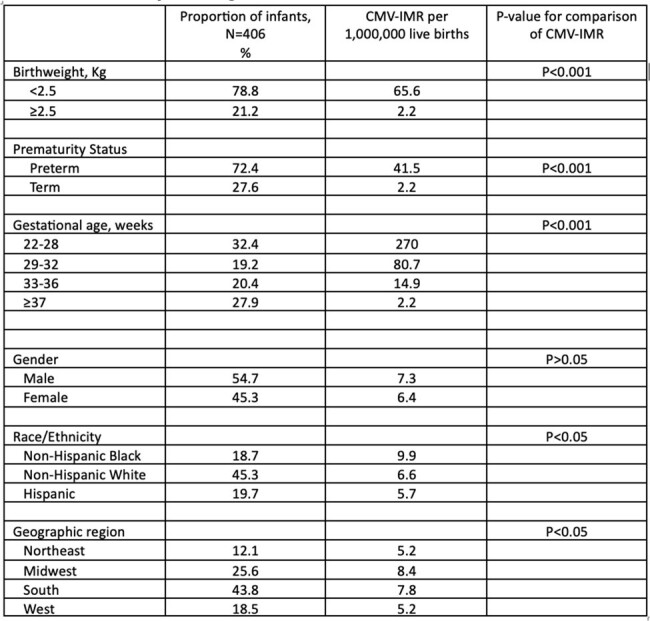
Characteristics of the national cohort of infants who died from CMV infections and their corresponding CMV-IMR, 2007-2009.

**Methods:**

This was a retrospective serial cross-sectional analysis of the CDC's linked birth and infant death records from 2007-2021. We used ICD-10 codes to identify infants < 1 year of age who had CMV infection listed as the underlying cause of death.The crude CMV-IMR was calculated per 1,000,000 live births and further stratified by gender, birth weight, gestational age, age at death, race, and census region. The Mann-Whitney U test was used to compare two groups and P < 0 .05 defined statistical significance. Trends were evaluated with Joinpoint regressionand expressed as average annual percentage change (AAPC) with 95% confidence intervals (CI).

**Results:**

Out of 59 million live births, 406 infant deaths were attributed to CMV infections (crude CMV-IMR of 6.9 per 1,000,000). This resulted in at least 30,044 years of potential life lost. Notable characteristics of the study cohort were (Table 1): 78.8% low birth weight, 72.4% preterm, 63.8% of the mothers had at least high school education, 14 states had 64% of the CMV deaths, and 45.3% died within 28 days of birth. The CMV-MR was higher in low birth weight and preterm infants and was inversely related to gestational age (Table 1). The CMV-IMR varied by race [highest in Non-Hispanic Black (9.9)], censusregion [highest in the Midwest (8.5)], and by state [4.3 in Illinoisand 12.3 in Alabama]. There was a non-significant decrease in the CMV-IMR from 9.3 to 4.1 (AAPC -2.9%; CI: -6.7 – 1.0%.)which was insignificant at 95%CI.

**Conclusion:**

The ubiquitous nature of CMV and the higher mortality in preterm and low birth weight infants should prompt serious consideration for early and universal CMV screening in these infants. The racial disparities in CMV-IMR suggest differential public health action toward awareness, prevention, and treatment of CMV. Addressing these disparities must be a top priority.

**Disclosures:**

All Authors: No reported disclosures

